# Supplementary material for: Yiai Fuzheng decoction inhibits triple-negative breast cancer by remodeling the immune microenvironment
Source: Front Immunol. 2025 Sep 30;16:1615631. doi: 10.3389/fimmu.2025.1615631 (PMC12518410; doi:10.3389/fimmu.2025.1615631)
Supplement: Supplementary file 2 [file DataSheet1.zip › Supplementary File 2/Treatment/D_vs_H/07_Pathway_Analysis/Pathway_Analysis/Sig_Pathways_Name/Aminoacyl-tRNA biosynthesis.pdf]

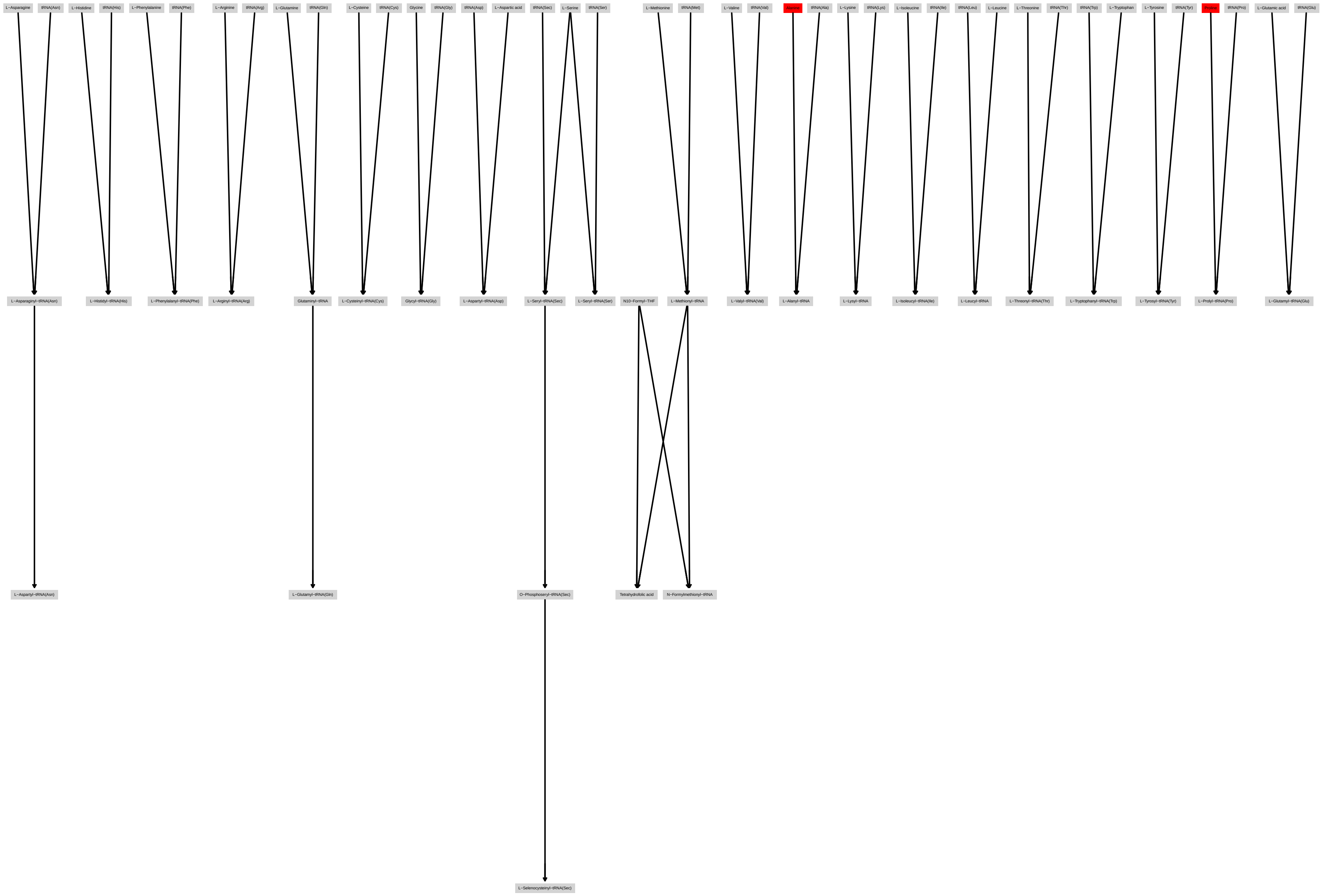
L−Asparagine
tRNA(Asn)
L−Histidine
tRNA(His)
L−Phenylalanine
tRNA(Phe)
L−Arginine
tRNA(Arg)
L−Glutamine
tRNA(Gln)
L−Cysteine
tRNA(Cys)
Glycine
tRNA(Gly)
tRNA(Asp)
L−Aspartic acid
tRNA(Sec)
L−Serine
tRNA(Ser)
L−Methionine
tRNA(Met)
L−Valine
tRNA(Val)
Alanine
tRNA(Ala)
L−Lysine
tRNA(Lys)
L−Isoleucine
tRNA(Ile)
tRNA(Leu)
L−Leucine
L−Threonine
tRNA(Thr)
tRNA(Trp)
L−Tryptophan
L−Tyrosine
tRNA(Tyr)
Proline
tRNA(Pro)
L−Glutamic acid
tRNA(Glu)
L−Asparaginyl−tRNA(Asn)
L−Histidyl−tRNA(His)
L−Phenylalanyl−tRNA(Phe)
L−Arginyl−tRNA(Arg)
Glutaminyl−tRNA
L−Cysteinyl−tRNA(Cys)
Glycyl−tRNA(Gly)
L−Aspartyl−tRNA(Asp)
L−Seryl−tRNA(Sec)
L−Seryl−tRNA(Ser)
N10−Formyl−THF
L−Methionyl−tRNA
L−Valyl−tRNA(Val)
L−Alanyl−tRNA
L−Lysyl−tRNA
L−Isoleucyl−tRNA(Ile)
L−Leucyl−tRNA
L−Threonyl−tRNA(Thr)
L−Tryptophanyl−tRNA(Trp)
L−Tyrosyl−tRNA(Tyr)
L−Prolyl−tRNA(Pro)
L−Glutamyl−tRNA(Glu)
L−Aspartyl−tRNA(Asn)
L−Glutamyl−tRNA(Gln)
O−Phosphoseryl−tRNA(Sec)
Tetrahydrofolic acid
N−Formylmethionyl−tRNA
L−Selenocysteinyl−tRNA(Sec)
